# Prediction Models for Risk of Cardiorespiratory Morbidity/Mortality and Fracture Among Young Adults With Cerebral Palsy

**DOI:** 10.1002/jcsm.13640

**Published:** 2025-04-03

**Authors:** Daniel G. Whitney, Edward A. Hurvitz

**Affiliations:** ^1^ Department of Physical Medicine and Rehabilitation University of Michigan Ann Arbor Michigan USA; ^2^ Institute for Healthcare Policy and Innovation University of Michigan Ann Arbor Michigan USA

**Keywords:** cardiorespiratory disease, cerebral palsy, fracture, frailty, risk prediction, sarcopenia

## Abstract

**Background:**

There is a dearth of screening tools for cardiorespiratory disease and fracture risk, such as risk prediction models, for adults with cerebral palsy (CP). There is heterogeneity of pathophysiology related to the severity of CP and aging, such that a suite of risk prediction models may be needed. Differentiating by sarcopenia versus frailty syndromes may be a useful, physiologic‐based framework to develop a suite of cardiorespiratory disease and fracture risk prediction models for adults with CP. The study objective was to determine if risk prediction models including widely available variables that are CP non‐specific and that may capture the physiologic components of frailty provide clinically useful prediction of salient health issues for young adults with CP.

**Methods:**

This retrospective cohort study used medical records from 01/01/2012 to 10/2/2022 from 18–40‐year‐olds with CP at a single Medical Centre. Logistic regression models were developed for three separate outcomes: 3‐year risk of respiratory morbidity/mortality, cardiovascular morbidity/mortality and fracture. The following predictors were included: age, sex, intellectual disabilities, epilepsy and four serum biomarkers (creatinine, glucose, calcium, carbon dioxide) from the clinical Basic/Comprehensive Metabolic Panel assay. Model performance measures were evaluated, including discrimination (c‐statistic) and calibration. Internal validation was assessed, and optimism‐corrected c‐statistics were computed using the bootstrap resampling method to assess model overfitting.

**Results:**

There were 805 young adults with CP with an average (SD) age of 26.5 (6.6) years and 47.8% were female. Over the 3‐year follow‐up, 24.6% had incident respiratory morbidity/mortality, 8.9% had incident cardiovascular morbidity/mortality and 7.0% had an incident fracture. The c‐statistic (95% CI) was 0.74 (0.70–0.78) for respiratory morbidity/mortality, 0.63 (0.57–0.70) for cardiovascular morbidity/mortality and 0.65 (0.58–0.73) for fracture. The optimism‐corrected c‐statistic was similar for respiratory morbidity/mortality (0.73) but lower for cardiovascular morbidity/mortality (0.58) and fracture (0.59), suggesting evidence of model overfitting. All models showed good calibration based on the slope of the predicted risk versus observed risk around 1.0 and the Hosmer–Lemeshow goodness‐of‐fit test, *P* = 0.305–0.903. However, the range of predicted risks was limited to ~20% for cardiovascular morbidity/mortality and ~55% for fracture, suggesting that these models have limited value for those with greater risk.

**Conclusions:**

Using widely available, CP non‐specific clinical variables, a well‐calibrated model was developed to predict 3‐year risk of respiratory morbidity/mortality among young adults with CP (discrimination, ~73%). The predictor set appeared less useful for predicting 3‐year risk of cardiovascular morbidity/mortality and fracture in this cohort.

## 1 Introduction

Adults with cerebral palsy (CP) have an excess and premature burden from fractures and cardiorespiratory morbidity and mortality [1, 2, 3, 4, 5, 6, 7, 8, 9, 10, 11, 12]. There is a ‘U’‐shaped association with the risk of respiratory morbidity/mortality across the adult lifespan, whereas the risk pattern of cardiovascular disease and fracture is relatively linear with increasing age for adults with CP [10]. The burden of disease for the adult population may be related to an interaction between age and severity of CP. Individuals with more severe forms of CP have a shorter lifespan where respiratory disease and bone fragility are common health issues within the young adult years, whereas individuals with milder forms of CP have a longer lifespan where respiratory disease, cardiovascular disease and bone fragility are common health issues within the middle‐aged to elderly years [10, 11, 12].

There are few, if any, screening tools for cardiorespiratory disease and fracture risk, such as risk prediction models, that are tailored to the different anatomy and physiology of adults with CP [13]. Given the heterogeneity of pathophysiology related to the severity of CP and aging, it seems unlikely that a single model can sufficiently capture varied disease risk profiles among the entirety of the adult population with CP. The field may be better served by a suite of risk prediction models. Ideally, risk prediction models include predictors that are prognostic, standardized and available to the intended end‐users. Many adults with CP receive care from clinicians who are unfamiliar with CP and the health changes over their life course [14, 15]. Risk prediction models will have greater widespread clinical adoption if they include measures that are CP non‐specific, as CP‐specific measures require specialized training and knowledge.

Differentiating by sarcopenia versus frailty syndromes may be a useful, physiologic‐based framework to begin developing a suite of cardiorespiratory disease and fracture risk prediction models for adults with CP. Sarcopenia describes a progressive age‐related loss of skeletal muscle mass and strength [16]. Frailty describes impairments of multiple physiological systems, including sarcopenia, associated with enhanced vulnerability to stressors [17]. Adults with milder forms of CP may develop muscle mass and strength to meet their functional needs for activities of daily living and may not exhibit the physiologic components of frailty. However, this subgroup is prone to accelerated age‐related loss of muscle mass and function [18]. Sarcopenia may better capture pathophysiologic mechanisms contributing to adverse health and function outcomes as adults with milder forms of CP age throughout their middle‐aged and elderly years. On the other hand, adults with more severe forms of CP may be better characterized by frailty because of several factors stemming from insufficient muscle development, dysfunction and multisystem impairments.

We recently examined if serum creatinine could serve as a prognostic biomarker for adults with CP to build into future risk prediction models [10]. We selected serum creatinine because it is a by‐product of muscle metabolism and associated with muscle mass [19] and may capture the varied frailty and sarcopenia syndromes across the age and CP severity spectrum. In our clinical cohort of adults ≥ 18 years old with CP (*n* > 1300), serum creatinine alone (i.e., no other predictors) displayed a promising predictive ability for 3‐year respiratory morbidity/mortality and fracture risk, but only among young adults [10]. Serum creatinine may have captured the underlying frailty in the young adult cohort but was not sufficient on its own to capture sarcopenia for the middle‐aged to elderly cohort. This is consistent with prior work suggesting that low serum creatinine levels are associated with frailty in non‐CP cohorts [20, 21]. These findings suggest the possibility of developing risk prediction models for young adults with CP from the framework of frailty via serum creatinine. Additional predictors in a model will likely enhance risk prediction.

Developing clinical risk prediction models must consider the usability of the intended end‐user to enhance clinical translation. Including one or more variables in a risk prediction model that are not consistently present in clinical settings can limit widespread clinical adoption as the model cannot be used without complete information on all included variables. Moreover, changing the variable's data form (e.g., value as continuous and categorized), creating dataset‐specific variable transformations (e.g., 4‐point age cut‐off based on spline modelling of the dataset) and including subjective measures may limit the potential for generalizability of the developed risk prediction model to other datasets (e.g., for external validation) and for clinical use (e.g., individualized risk estimation).

The objective of this study was to determine if risk prediction models composed of serum creatinine plus variables that capture other physiologic components of frailty provide clinically useful prediction of salient health issues for young adults (18–40 years old) with CP, including 3‐year risk of respiratory morbidity/mortality, cardiovascular morbidity/mortality and fracture. The 3‐year risk window was selected as it provides a reasonable length of time to assess a patient's risk and then clinically act upon the result (e.g., further diagnostics, specialty referral and implementing prevention strategies). The selection of predictors was based on the motivation to develop clinically friendly models with a high potential for generalizability. Many factors were weighed for selecting predictors and their form (e.g., value as continuous and categorized), such as the predictor's relevance to frailty and prognostication, feasibility by the broad clinical community caring for adults with CP and limiting the number and transformation of predictors to avoid model overfitting.

The final list of possible predictors included age, sex, intellectual disabilities, epilepsy and four serum biomarkers: creatinine, glucose, calcium and carbon dioxide. Co‐occurring intellectual disabilities and epilepsy were included as they are associated with increasing the medical complexity of the individual with CP [22] and are associated with an increased risk of cardiorespiratory morbidity/mortality and fracture [23, 24]. The four serum biomarkers were selected as they are part of the Basic and Comprehensive Metabolic Panel that are objective measures, analysed using standardized methods and are widely available clinically for routine measurement. Further, these biomarkers may collectively represent various physiological systems that are relevant to the frailty sequela. For example, glucose may reflect impaired metabolic, muscle and lipid functioning [25, 26, 27]. Calcium may reflect bone metabolism, endocrine effects (e.g., impaired parathyroid function) or malnutrition. Carbon dioxide is a by‐product of cellular metabolism that can indicate issues with regulating blood acid–base balance or respiratory function.

## 2 Methods

### 2.1 Data Source and Cohort Selection

This was a retrospective cohort study that accessed electronic medical records from a single Medical Centre in Southeast Michigan from 01/01/2012 to 10/02/2022, as previously described [10]. To be included in this study, individuals had a confirmed diagnosis of CP, had a Basic or Comprehensive Metabolic Panel laboratory assessment between 01/01/2013 (to allow for a 1‐year lookback period) and 10/3/2019 (to allow for a 3‐year follow‐up) and were 18–40 years old by the date of the laboratory assessment. The start of follow‐up (i.e., day 0) was the first eligible laboratory assessment where all biomarkers were obtained from the same blood draw. Further inclusion for analysis involved ≥ 1 year of evidence of being treated at our Medical Centre prior to their Day 0 [10]. This 1‐year lookback period was used to identify if outcome events occurred prior to or on Day 0, which resulted in exclusion from that analysis as described below. Of the 1518 adults ≥ 18 years old with ≥ 1 laboratory assessment, 276 were excluded because of < 1 year of a lookback period (18.2%), 21 because of excessively high serum biomarkers that included creatinine or glucose (not calcium or carbon dioxide) defined as four standard deviations above the mean of the entire cohort (1.7%) and 362 as they were older than 40 years of age by day 0 (31.0%). The University's Institutional Review Board approved this study, and patient consent was waived as this study involved no more than minimal risk.

### 2.2 Outcomes

The three outcomes included a binary indication (yes/no) for incident respiratory morbidity/mortality, cardiovascular morbidity/mortality and fracture from Day 1 to 3 years after Day 0. To enhance clinical relevance, cardiorespiratory morbidities included conditions that have a significant and sudden consequential effect on the patient's health and well‐being. Respiratory morbidity included respiratory failure or pneumonia, whichever came first, which is a leading cause of death for adults with CP [3]. Cardiovascular morbidity included myocardial infarction, heart failure or cerebrovascular disease, whichever came first. Cardiorespiratory mortality was determined by any respiratory and cardiovascular indication as to the cause of death, as previously described [10]. Fracture included a clinician‐defined ‘pathological’ fracture at any site (presumed to reflect a fragility fracture) or all‐cause fracture of the vertebral column, hip, lower extremities, humerus, forearm or multiple sites, but not at ‘minor’ (all‐cause fracture at thoracic sites or clavicle), unidentifiable or skull sites.

To identify new events as opposed to conditions that occurred prior to or on Day 0 being cared for after Day 0, specific conditions that occurred during the 3‐year follow‐up were counted if there was no evidence of that same condition during the 1‐year lookback period. For pneumonia, a 30‐day lookback period was used, as opposed to the 1‐year period, to rule out follow‐up pneumonia, as this condition may be more likely to occur multiple times in this population.

### 2.3 Predictors

The list of predictors included age (continuous), sex (female/male), intellectual disabilities (yes/no), epilepsy (yes/no) and four serum biomarkers (continuous): creatinine, glucose, calcium and carbon dioxide. Age by Day 0, sex and co‐occurring intellectual disabilities and epilepsy were retrieved from the medical records. The four serum biomarkers were collected from the same blood draw and analysed using the Basic or Comprehensive Metabolic Panel assays.

### 2.4 Statistical Analysis

Baseline characteristics of the predictors, race/ethnicity, primary insurance and start of the follow‐up year and the 3‐year risk of each outcome were summarized for the cohort.

Risk prediction models were developed using logistic regression for each outcome separately. To enhance generalizability, the eight predictors were included in each model as main effects only (i.e., no interactions). The regression coefficient and odds ratio (OR) with 95% confidence intervals (CIs) was estimated for each predictor. To assess the performance of the model, discrimination and calibration were assessed. Discrimination refers to the predictive ability of the model to accurately distinguish between those that did and did not develop the outcome. The extent of discrimination was assessed using the c‐statistic, which ranges from 0.50 (equivalent to random guessing) to 1.00 (perfect predictive ability) [28]. Calibration refers to the extent that the predicted probability from the model matches the observed frequencies in the dataset. Calibration was assessed visually by the calibration plot, the value of the slope between the predicted versus observed risk (where a slope of 1.00 indicates perfect calibration) and using the Hosmer–Lemeshow goodness‐of‐fit test. Internal validation of each model was assessed to provide evidence for potential overfitting and ‘optimism’ (a form of overestimation bias) in the model's performance by estimating the optimism‐corrected c‐statistic using the bootstrap resampling method with 2000 samples. The bootstrap approach has been shown to be superior compared to other approaches (e.g., split‐sample and cross‐validation) for internal validation of predictive logistic regression models [29].

### 2.5 Sensitivity Analysis

To provide a secondary model that limits overfitting, the primary models were developed using Firth's penalized likelihood [30], which is a shrinkage estimation method that ‘shrinks’ the regression coefficient towards zero.

### 2.6 Sample Size Estimation

We followed recommendations by van Smeden et al. [31, 32] and Riley et al. [33, 34] using a four‐step procedure to estimate the sample size needed based on eight predictors, the incidence of each outcome in this study's cohort and all other recommendations [35]. The results of the four‐step procedure to estimate the sample size needed to mitigate model overfitting were up to *n* = 679. This study developed models with a larger sample size, thus mitigating the potential for major overestimation bias based on these parameters.

### 2.7 Exploratory Analysis

We performed exploratory analyses that assessed for interactions between serum creatinine with age, sex and each biomarker separately, as it was unknown if there is effect modification by these variables in this population. Where there was evidence of effect modification, models were developed that included the main and interaction term and adjusted for the other predictors as main effects. The effect estimates of the interaction term and the model's c‐statistic and calibration were assessed. In these exploratory analyses, it was determined a priori not to adjust the *P*‐value or CIs for multiple testing [36, 37, 38]. Findings should be interpreted as hypothesis‐generating.

Analyses were performed using SAS version 9.4 (SAS Institute, Cary, NC, USA), and *P* ≤ 0.05 (two‐tailed) was considered statistically significant.

## 3 Results

Baseline descriptive characteristics and laboratory values from the 18–40‐year‐old cohort with CP (*n* = 805) are presented in Table 1. Notably, the median and interquartile range for each of the four serum biomarkers was within ‘normal’ ranges. Over the 3‐year follow‐up, 198 of the 805 (24.6%) had incident respiratory morbidity/mortality (19.5% pneumonia, 4.8% respiratory failure, 0.6% respiratory‐related mortality), 72 of the 805 (8.9%) had incident cardiovascular morbidity/mortality (6.3% cerebrovascular disease, 1.0% congestive heart failure, 0.6% myocardial infarction, 1.0% cardiovascular‐related mortality), and 56 of 797 without a baseline fracture (7.0%) had an incident fracture.

**TABLE 1:** Baseline descriptive characteristics of 18–40‐year‐olds with cerebral palsy (*n* = 805).

	% (*n*)
Age, mean (standard deviation)	26.5 (6.6)
Sex	
Female	47.8 (385)
Male	52.2 (420)
Race	
Black	14.4 (116)
White	78.9 (635)
Other	6.7 (54)
Primary insurance	
Medicaid	50.7 (408)
Medicare	15.7 (126)
Private	25.2 (203)
Other	0.9 (7)
Missing	7.6 (61)
Start year	
2013	21.2 (171)
2014	18.4 (148)
2015	13.9 (112)
2016	12.2 (98)
2017	11.3 (91)
2018	11.4 (92)
2019	11.6 (93)
Intellectual disabilities	22.5 (181)
Epilepsy	51.8 (417)
**Outcomes in 1‐year baseline period**	
Pneumonia	
0–14 days prior	0.9 (7)
15–30 days prior	0.8 (6)
31–90 days prior	2.5 (20)
91–365 days prior	1.5 (12)
Respiratory failure	1.0 (8)
Cerebrovascular disease	2.7 (22)
Heart failure	0.3 (2)
Myocardial infarction	0.1 (1)
Fracture	
Major	1.0 (8)
Minor	0.3 (2)
Skull	0.3 (2)
**Laboratory values, median (interquartile range)**	
Creatinine, mg/dL	0.64 (0.50–0.80)
Glucose, mg/dL	88 (80–99)
Calcium, mg/dL	9.5 (9.2–9.8)
Carbon dioxide, mmol/L	27.0 (25.0–29.0)

### 3.1 Association Between Predictors and Outcomes and Model Performance

**FIGURE 1:**
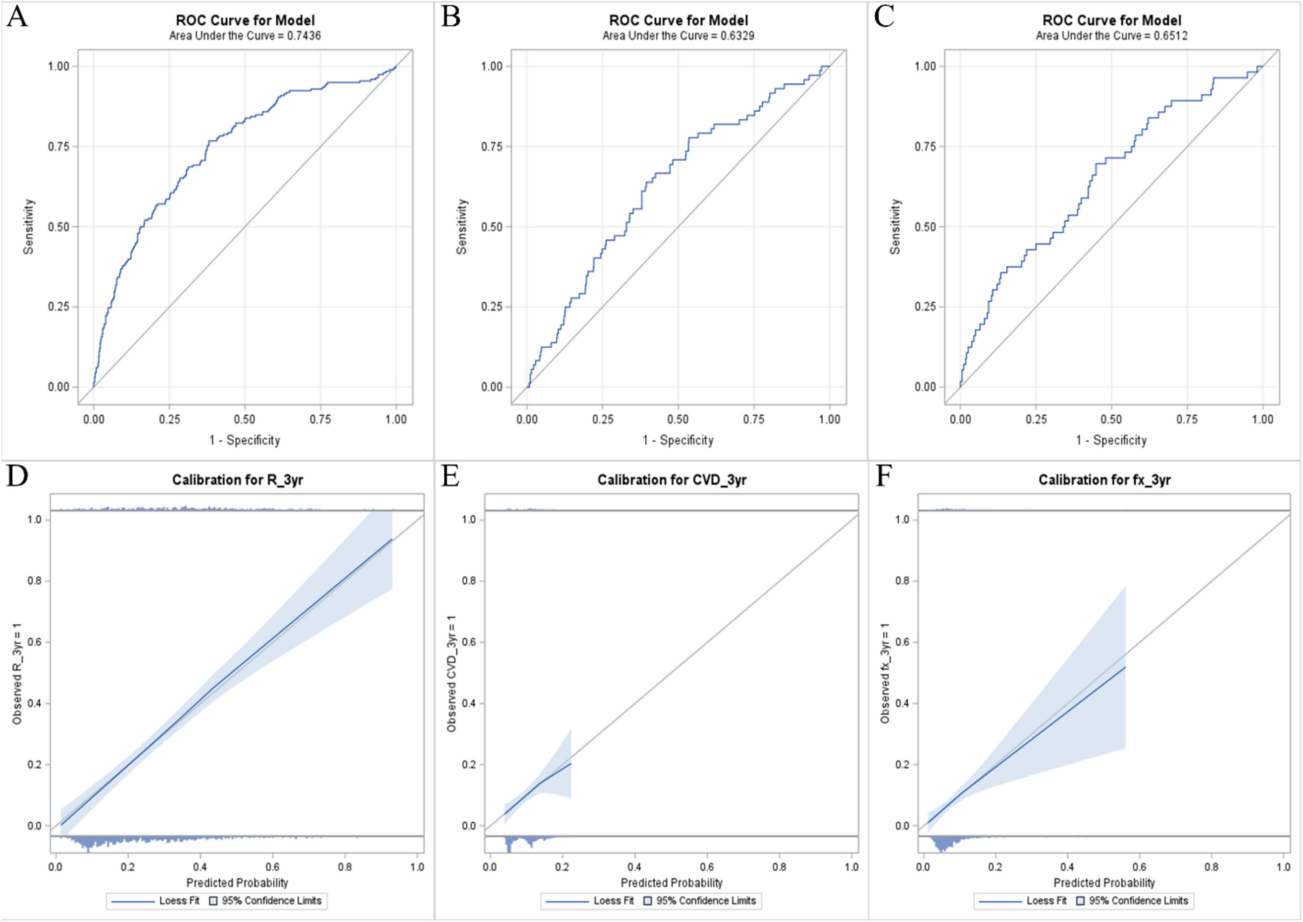
Panels (A–C) displays the receiver operating characteristic curve (area under the curve is numerically similar to the c‐statistic), and Panels (D–F) represent the calibration plot for the final models predicting 3‐year risk of (A,D) respiratory morbidity/mortality (R_3yr), (B,E) cardiovascular morbidity/mortality (CVD_3yr) and (C,F) fracture (fx_3yr) among young adults with cerebral palsy.

The OR and performance measures of outcomes including all eight predictors per model are presented in Table 2. The receiver operating characteristic curve and calibration plot for each model are shown in Figure 1. The OR of each predictor was similar in the model with and without shrinkage estimation. The c‐statistic (95% CI) of the full model was 0.74 (0.70–0.78) for respiratory morbidity/mortality, 0.63 (0.57–0.70) for cardiovascular morbidity/mortality and 0.65 (0.58–0.73) for fracture. The optimism‐corrected c‐statistic was similar for respiratory morbidity/mortality (0.73) suggesting little evidence of model overfitting, but lower for cardiovascular morbidity/mortality (0.58) and fracture (0.59) suggesting evidence of model overfitting. All models showed good calibration based on the slope of the predicted risk versus observed risk around 1.0. However, the range of predicted risks was limited to ~0.20 for cardiovascular morbidity/mortality and ~0.55 for fracture. This suggests that these models have limited risk prediction across a range of risks and may not be a useful model as is. Therefore, the regression coefficients for risk prediction were not reported for cardiovascular morbidity/mortality or fracture.

**TABLE 2:** Odds ratio (OR) of each outcome and performance measures for 18–40‐year‐olds with cerebral palsy (*n* = 805).

	Respiratory morbidity/mortality	Cardiovascular morbidity/mortality	Fracture
	OR (95% CI)	OR (95% CI)	OR (95% CI)
**No shrinkage**			
Age (per 5 years)	0.85 (0.74, 0.98)	0.98 (0.80, 1.18)	1.17 (0.94, 1.44)
Sex (reference: female)	1.42 (0.98, 2.07)	0.94 (0.55, 1.59)	0.93 (0.51, 1.68)
Intellectual disabilities	1.73 (1.17, 2.57)	1.42 (0.82, 2.44)	1.24 (0.66, 2.32)
Epilepsy	2.85 (1.95, 4.15)	2.32 (1.34, 4.02)	1.39 (0.78, 2.49)
Creatinine (per 0.10 mg/dL)	0.84 (0.77, 0.92)	1.01 (0.91, 1.12)	0.93 (0.82, 1.06)
Glucose (per 10 mg/dL)	1.24 (1.13, 1.35)	1.03 (0.91, 1.16)	1.18 (1.05, 1.33)
Calcium (per 0.2 mg/dL)	0.94 (0.87, 1.00)	0.97 (0.89, 1.05)	0.93 (0.86, 1.02)
Carbon dioxide (per 1 mmol/L)	1.08 (1.03, 1.14)	1.03 (0.96, 1.11)	1.06 (0.97, 1.15)
Model c‐statistic (95% CI)	0.74 (0.70, 0.78)	0.63 (0.57, 0.70)	0.65 (0.58, 0.73)
Model optimism‐corrected c‐statistic (corrected factor)	0.73 (0.01)	0.58 (0.05)	0.59 (0.06)
Slope (SE) of predicted vs. observed	1.03 (0.09)	0.99 (0.26)	0.99 (0.20)
Hosmer/Lemeshow GOF *P*‐value	0.305	0.903	0.335
**Shrinkage**			
Age (per 5 years)	0.85 (0.74, 0.98)	0.98 (0.81, 1.18)	1.16 (0.95, 1.43)
Sex (reference: female)	1.41 (0.97, 2.06)	0.93 (0.56, 1.56)	0.92 (0.52, 1.65)
Intellectual disabilities	1.72 (1.17, 2.55)	1.43 (0.84, 2.43)	1.25 (0.68, 2.31)
Epilepsy	2.80 (1.93, 4.08)	2.29 (1.34, 3.91)	1.39 (0.79, 2.44)
Creatinine (per 0.10 mg/dL)	0.85 (0.78, 0.92)	1.01 (0.92, 1.12)	0.94 (0.83, 1.06)
Glucose (per 10 mg/dL)	1.23 (1.13, 1.35)	1.03 (0.92, 1.16)	1.18 (1.06, 1.33)
Calcium (per 0.2 mg/dL)	0.94 (0.89, 1.00)	0.96 (0.89, 1.05)	0.93 (0.86, 1.01)
Carbon dioxide (per 1 mmol/L)	1.08 (1.03, 1.14)	1.03 (0.96, 1.11)	1.06 (0.97, 1.14)

*Note:* Shrinkage estimation used the Firth penalized likelihood method.

Abbreviations: CI, confidence interval; GOF, goodness of fit; SE, standard error.

The regression coefficients from the final model with and without shrinkage estimation to calculate an individual's predicted 3‐year risk of respiratory morbidity/mortality are presented in Table 3 along with an example of how to use the model.

**TABLE 3:** Model for 3‐year risk of respiratory morbidity/mortality among 18–40‐year‐olds with cerebral palsy (*n* = 805).

	No shrinkage	Shrinkage
	Regression coefficient	Regression coefficient
Intercept	−1.4580	−1.4340
Age	−0.0321	−0.0316
Sex	0.3516	0.3466
Intellectual disabilities	0.5496	0.5447
Epilepsy	1.0460	1.0311
Creatinine	−1.7175	−1.6781
Glucose	0.0212	0.0208
Calcium	−0.2969	−0.2928
Carbon dioxide	0.0793	0.0779

*Note:* To calculate the individual 3‐year risk of respiratory morbidity/mortality, first calculate the linear predictor as follows: where *b* = regression coefficient: Intercept + (*b*_age_ × age) + (*b*_sex_ × [1 if male, 0 if female]) + (*b*_intellectual disabilities_ × [1 if present, 0 if absent]) + (*b*_epilepsy_ × [1 if present, 0 if absent]) + (*b*_creatinine_ × creatinine value) + (*b*_glucose_ × glucose value) + (*b*_calcium_ × calcium value) + (*b*_carbon dioxide_ × carbon dioxide value). Then, calculate the risk as follows: 1/(1 + exp(−(linear predictor))). For example, for a 24.4‐year‐old male with CP with co‐occurring epilepsy and lab values of 0.56 mg/dL for creatinine, 91 mg/dL for glucose, 9.8 mg/dL for calcium and 27 mmol/L for carbon dioxide, the linear predictor from the nonshrinkage model would be as follows: −1.458 + (*b*_age_ × 24.4) + (*b*_sex_ × 1) + (*b*_intellectual disabilities_ × 0) + (*b*_epilepsy_ × 1) + (*b*_creatinine_ × 0.56) + (*b*_glucose_ × 91) + (*b*_calcium_ × 9.8) + (*b*_carbon dioxide_ × 27) = −0.6448. The risk would then be 1/(1 + exp(0.6448)) = 0.3442 or 34.42%.

### 3.2 Exploratory Analysis

When predicting 3‐year risk of respiratory morbidity/mortality, there was evidence of an interaction by creatinine for glucose (*P* for interaction, 0.004) and calcium (*P* for interaction, < 0.001). The OR per unit increase in glucose and calcium conditional on creatinine level (as 10^th^, 25^th^, 50^th^, 75^th^ and 90^th^ percentile) is presented in Table 4. A brief interpretation is that at higher creatinine levels, higher glucose levels were associated with increased risk, whereas the opposite pattern was observed for calcium. Compared with the primary model without interactions, the model that included the main and interaction effects for creatinine by glucose and creatine by calcium had similarly excellent model calibration and a negligible increase in the c‐statistic before (0.76; 95% CI = 0.72–0.80) and after (0.74; corrected factor = 0.02) correcting for optimism.

**TABLE 4:** Results of effect modification among 18–40‐year‐olds with cerebral palsy (*n* = 805).

	Respiratory morbidity/mortality	Fracture
	OR (95% CI)	OR (95% CI)
Glucose (per 10 mg/dL)		
Creatinine, 0.38 mg/dL	1.16 (1.03, 1.30)	—
Creatinine, 0.50 mg/dL	1.19 (1.08, 1.31)	—
Creatinine, 0.64 mg/dL	1.23 (1.13, 1.35)	—
Creatinine, 0.80 mg/dL	1.28 (1.15, 1.42)	—
Creatinine, 0.95 mg/dL	1.33 (1.16, 1.52)	—
Calcium (per 0.2 mg/dL)		
Creatinine, 0.38 mg/dL	0.99 (0.92, 1.07)	—
Creatinine, 0.50 mg/dL	0.96 (0.90, 1.03)	—
Creatinine, 0.64 mg/dL	0.93 (0.87, 0.99)	—
Creatinine, 0.80 mg/dL	0.89 (0.82, 0.97)	—
Creatinine, 0.95 mg/dL	0.86 (0.77, 0.95)	—
Creatinine (per 0.10 mg/dL)		
Age, 18.8 years	—	0.73 (0.59, 0.91)
Age, 20.5 years	—	0.76 (0.62, 0.93)
Age, 25.3 years	—	0.85 (0.74, 0.99)
Age, 31.4 years	—	0.99 (0.86, 1.13)
Age, 36.9 years	—	1.13 (0.94, 1.35)

*Note:* Each model includes the main and interaction effect displayed in the table and the main effect of the other predictors.

Abbreviations: CI, confidence interval; OR, odds ratio.

When predicting 3‐year risk of cardiovascular morbidity/mortality, there was no strong evidence of an interaction (all *P* for interaction, > 0.170).

When predicting 3‐year risk of fracture, there was evidence of an interaction by age for creatinine (*P* for interaction, 0.005). The OR per unit increase in creatinine conditional on age (as 10^th^, 25^th^, 50^th^, 75^th^ and 90^th^ percentile) is presented in Table 4. A brief interpretation is that at younger ages, higher creatinine levels were associated with decreased risk. Compared with the primary model without interactions, the model that included the main and interaction effects for creatinine by age had a slightly worse calibration slope (slope = 1.10) but improvement in the model calibration range (predicted risks up to ~0.65) and the c‐statistic before (0.67; 95% CI = 0.59–0.74) and after (0.61; corrected factor = 0.06) correcting for optimism.

## 4 Discussion

This study used widely available, CP non‐specific clinical variables to develop and assess the performance of risk prediction models for young adults with CP. One main outcome of this study was the development of a well‐calibrated model to predict 3‐year risk of respiratory morbidity/mortality among young adults with CP with a discrimination of ~73%. This study helps contribute one to a suite of risk prediction models for capturing the risk of salient health outcomes for adults with CP. However, external validation in different cohorts is needed to assess generalizability and refine the model if needed to optimize for widespread clinical use.

An important finding was that the predictor set (as main effects) was less useful for predicting 3‐year risk of cardiovascular morbidity/mortality and fracture. Cardiovascular disease and fracture are more common health issues for young adults with versus without CP [39], and accurate screening tools for these outcomes could assist clinical detection and resultant prevention strategies. However, respiratory issues are more problematic for young adults with CP. For example, ~25% of the young adult cohort with CP developed respiratory morbidity/mortality over the 3‐year period, as compared with ~9% for cardiovascular morbidity/mortality and ~7% for fracture. Therefore, the risk prediction model developed in this study for 3‐year risk of respiratory morbidity/mortality has high clinical priority. Nevertheless, future work is needed to identify other possible predictors or methods to capture the risk of cardiovascular morbidity/mortality and fracture for young adults with CP, such as bone biomarkers, standardized nutritional assessment variables and renal function, which may help to better understand if the extent of serum creatinine is due to chronically low muscle mass, cachexia or muscle wasting, nutritional status or declining renal function. Additionally, relevant measures quantifying the muscle‐bone unit [40], such as from clinical DXA scans, may enhance the prognostication of fracture, whereas measures of inflammation (e.g., CRP) may enhance the prognostication of cardiovascular outcomes. Other measures warrant further examination, such as prognostication by socioeconomic status, blood pressure, inflammation and anthropometrics.

The exploratory analysis found that the relationship between age and some biomarkers with respiratory morbidity/mortality and fracture was modified by creatinine. However, model performance measures (e.g., c‐statistic) including the interaction terms did not meaningfully change when predicting these outcomes 3‐year risk of respiratory morbidity/mortality. As this study is geared towards prognostication, discussion on physiologic associations and speculation on underlying causality is beyond the scope of this work. These findings are also subject to biased effect estimates and associations given the exploratory nature of the analysis and the lack of adjustment for multiple testing. Multiple testing adjustment is often recommended for confirmatory studies to reduce the possibility of a familywise error rate [36]. This is because *P*‐values and the resulting CIs are often used to indicate how replicable an association is from a particular dataset [38]. However, accounting for familywise error is less relevant for data exploration [36, 37, 38]. Taken together, the findings from the exploratory analysis should be interpreted as hypothesis‐generating as opposed to confirmation of associations.

Two of the major downfalls of translating risk prediction models to clinical practice are clinical feasibility and how well the model generalizes, or ‘works’, for individuals/cohorts outside of the dataset in which the model was developed. From a logistical standpoint, we selected variables that clinics have access to, including biomarkers that can be obtained from routine, standardized assays and avoided variables that would require unique training and knowledge in CP to measure. This allows for greater inclusion of the intended end‐users to use the models (e.g., physicians caring for adults with CP), enhancing clinical feasibility. From a data analytic standpoint, overfitting models can limit generalizability, for example, by introducing complex interactions or data‐driven categorization of variables, such that these decisions are dataset‐specific and may not hold for external datasets and situations. This study kept the predictor variables in their raw form, that is, no data transformation such as categorization or log‐transformed. We also assessed the possible extent of overfitting by computing the optimism‐corrected c‐statistic and found little evidence of overfitting for the model predicting respiratory morbidity/mortality.

However, even if post‐collection methodological decisions are reasonably balanced, the models are only as good as the data collected and the level of knowledge of data representation. There are limitations of secondary data analysis of electronic medical records from a single site, especially as it relates to interpreting and translating risk prediction models. It is unknown how representative our cohort is to the greater young adult population with CP. Although we sampled over several years, our cohort may be biased towards an unhealthier or more socioeconomically advantaged subgroup as we obtained information based on clinical encounters. Further, we included individuals if they had an eligible Basic or Comprehensive Metabolic Panel assessed for the biomarkers, which may introduce sample selection bias. In our prior study that included adults ≥ 18 years old, in which this study's young adult cohort was derived, 42% of the *n* = 3425 adults with CP at our Medical Centre had these biomarkers measured [10]. As these biomarkers are generally assessed for routine or preoperative screening, the study findings have a reasonable probability of representing the full adult cohort with CP seen at our Medical Centre. However, the indication for these blood draws cannot be obtained from the records. If the indication(s) for biomarker assessment is related to patient characteristics, there are situations that can confound or modify the examined associations and performance measures. At the lowest generalizable end, study findings can be clinically useful for young adult patients with CP that would normally have their biomarkers assessed as part of their routine care but should be interpreted with caution for patients with CP that would undergo blood draw for biomarker assessment for nonroutine reasons due to the possibility of sample selection bias.

It is important to note that secondary data analysis of electronic medical records from a single site has strengths regarding the practicality of conducting health‐related research for adults with CP. One main strength is the acquisition of a large, longitudinal cohort of adults with CP with clinical variables and biomarkers collected from standardized clinical assays. There are a limited number of opportunities to conduct large‐scale health‐related research for adults with CP, leaving clinicians with little information to inform decision‐making. Large datasets for health‐related research for individuals with CP are either not publicly available to researchers (e.g., international registries), include children only, are cross‐sectional (e.g., National Survey of Children's Health) or do not contain biomarker data (e.g., insurance claims). Further, large‐scale prospective cohort studies are time intensive, incredibly costly and impractical. Secondary data analysis of real‐world clinical datasets is a feasible option and is reasonable, with appropriate interpretations, given the lack of current research.

There are other limitations that may impact the interpretation of study findings. It was not possible to identify if the blood draw occurred following fasting or the status of the patient's hydration or diet, which can impact the biomarkers. Loss to follow‐up and appropriate right‐censoring are important for regression modelling and risk prediction but can be challenging to accurately assess using electronic medical records. To mitigate this bias, individuals were included if they had evidence of treatment at our Medical Centre in the baseline period. Anthropometric measures, such as height and body mass, and CP‐specific measures, such as the gross motor function classification system or topographical distribution of affected areas, were intentionally not examined in this study but may help to describe the cohort further to assess generalizability. However, the medical records are inconsistent in containing these measures, in reporting how these measures were obtained, and it is common for a prior value to be ‘carried forward’ without remeasuring. This study examined risks up to 3 years, which may not be enough time for certain outcomes, such as cardiovascular events. Future studies should consider longer follow‐up periods to develop prognostic models. Further, as this study did not access information regarding nutritional status and renal function, it is unknown if the serum creatinine generally reflected chronic low muscle mass common to people with CP, cachexia or muscle wasting, nutrition status or changes in kidney function and how this may have impacted the risk prediction models.

Using a frailty‐based framework helped to identify possible predictors to develop risk prediction models for 3‐year risk of salient health issues for young adults with CP. This study developed a well‐calibrated risk prediction model for respiratory morbidity/mortality using widely available clinical variables for young adults with CP that underwent blood draw for biomarker assessment at our Medical Centre. The predictor set was less useful for predicting the risk of cardiovascular morbidity/mortality and fracture in this cohort. Future development of a suite of risk prediction models to inform disease detection and prevention may need to consider a variety of possible predictors for high‐priority health outcomes.

## Ethics Statement

The manuscript complies with the ethical guidelines for authorship and publishing in the Journal of Cachexia, Sarcopenia and Muscle. The study has been approved by the appropriate ethics committee and has been performed in accordance with the ethical standards laid down in the 1964 Declaration of Helsinki and its later amendments. The University's Institutional Review Board approved this study, and patient consent was waived as this study involved no more than minimal risk.

## Conflicts of Interest

Dr Whitney has received consulting fees from Rifton and compensation fees for serving on review panels of federal agencies, all of which are unrelated to the present work.
